# Krill Oil Ameliorates Mitochondrial Dysfunctions in Rats Treated with High-Fat Diet

**DOI:** 10.1155/2015/645984

**Published:** 2015-08-02

**Authors:** Alessandra Ferramosca, Annalea Conte, Vincenzo Zara

**Affiliations:** Dipartimento di Scienze e Tecnologie Biologiche ed Ambientali, Università del Salento, Via Provinciale Lecce-Monteroni, 73100 Lecce, Italy

## Abstract

In recent years, several studies focused their attention on the role of dietary fats in the pathogenesis of hepatic steatosis. It has been demonstrated that a high-fat diet is able to induce hyperglycemia, hyperinsulinemia, obesity, and nonalcoholic fatty liver disease. On the other hand, krill oil, a novel dietary supplement of n-3 PUFAs, has the ability to improve lipid and glucose metabolism, exerting possible protective effects against hepatic steatosis. In this study we have investigated the effects of krill oil on mitochondrial energetic metabolism in animals fed a high-fat diet. To this end, male Sprague-Dawley rats were divided into three groups and fed for 4 weeks with a standard diet (control group), a diet with 35% fat (HF group), or a high-fat diet supplemented with 2.5% krill oil (HF+KO group). The obtained results suggest that krill oil promotes the burning of fat excess introduced by the high-fat diet. This effect is obtained by stimulating mitochondrial metabolic pathways such as fatty acid oxidation, Krebs cycle, and respiratory chain complexes activity. Modulation of the expression of carrier proteins involved in mitochondrial uncoupling was also observed. Overall, krill oil counteracts the negative effects of a high-fat diet on mitochondrial energetic metabolism.

## 1. Introduction

Nonalcoholic fatty liver disease (NAFLD) is a condition in which fat accumulates in the cells of liver. It occurs when the rate of hepatic fatty acid uptake from plasma and* de novo* lipogenesis is greater than the rate of fatty acid oxidation and export. Traditionally, NAFLD is a histological disease which progresses from pure fatty liver (simple steatosis) through nonalcoholic steatohepatitis (NASH) to liver fibrosis, cirrhosis, and eventually hepatocellular carcinoma.

Several evidences indicate that hepatic mitochondrial dysfunction is crucial to the pathogenesis of NAFLD [1–3]. However, the whole spectrum of mitochondrial adaptations during hepatic steatosis remains to be characterized. A recent study [4] proposed a model describing the development of NAFLD in which overflow of free fatty acids to hepatocytes leads to an increase in mitochondrial energetic metabolism. This seems necessary to supply the energy needed for triglycerides storage. Later, mitochondrial oxidative stress, morphological abnormalities, and finally hepatocytes apoptosis lead to the development of the second hit of steatosis.

As liver fat accumulation can originate from dietary intake, it is of critical importance to understand how different diets and their macronutrient composition can influence the development of NAFLD. Despite contradictory results regarding the role of different diets on NAFLD, it is reasonable to propose that overconsumption of either fat or carbohydrates may promote the development of NAFLD [5]. It seems that specific fatty acids or carbohydrates are more prone to induce or improve NAFLD. Indeed, diets rich in fatty acids, particularly saturated, or in refined carbohydrates, can actually exacerbate NAFLD [5].

Interestingly, it has been demonstrated that dietary fats not only influence the pathogenesis of NAFLD or NASH, but also may prevent or reverse its expression [6]. Therefore, a condition of fatty liver was induced in rodent animal models by the use of high-fat (HF) diets [2, 7] whereas supplementation of the diet with n-3 PUFAs helped in the prevention and/or in the treatment of steatotic liver [2]. Moreover, there is much stronger evidence that polyunsaturated oils are responsible for the progression from simple steatosis to NASH and these effects may result from a high intake of total PUFAs or may result from a high ratio of n-6 to n-3 fatty PUFAs [8].

In this context, it has been reported that a dietary krill oil (KO) supplementation has the capacity to reduce fatty liver in mice and rats [2, 9, 10]. Recently, it has been also demonstrated that this oil stimulated the catabolization of fat excess introduced by hypercaloric diets, while inhibiting* de novo* fatty acid synthesis and therefore preventing the onset of fatty liver [2, 10].

KO is a novel dietary supplement extracted from Antarctic krill (*Euphausia superba*), a shrimp-like zooplankton, which has become increasingly popular during the last decade. This oil contains two n-3 PUFAs, eicosapentaenoic acid (EPA, 20 : 5) and docosahexanoic acid (DHA, 22 : 6), in amounts similar to those present in fish oil. However, in KO these long-chain n-3 fatty acids are present in the form of phospholipids rather than triglycerides as in the case of fish oil [11]. Furthermore, the ratio of EPA to DHA present in KO is higher than that in fish oil. It has been proposed that these peculiar characteristics of KO composition render it more efficient than fish oil in the modulation of activity and expression of many enzymes involved in lipid metabolism in animal models [10, 12].

In this study we have investigated the molecular mechanism by which KO exerts a possible protective effect on mitochondrial energetic metabolism in animals fed a diet enriched in fat. To this end, we have thoroughly analyzed mitochondrial respiration by monitoring oxygen consumption and the activity of the single respiratory chain complexes in mitochondria. The expression of proteins related to the respiratory chain function was also investigated. The obtained results led us to depict a possible framework for the protective effects of KO against hepatic steatosis.

## 2. Materials and Methods

### 2.1. Materials

Bio-Rad protein assay kit was purchased from Bio-Rad; sodium pyruvate, malic acid, succinic acid, ascorbic acid,* N*,*N*,*N*′,*N*′-tetramethyl-*p*-phenylenediamine (TMPD), palmitoyl-L-carnitine, rotenone, antimycin A, oligomycin, coenzyme A trilithium salt (CoA-SH), acetyl-CoA, oxaloacetic acid, thiamine pyrophosphate (TPP), 5,5-dithiobis-(2-nitrobenzoic acid) (DTNB), 2,6-dichlorophenolindophenol (DCPIP), carbonyl cyanide-4-(trifluoromethoxy)phenylhydrazone (CCCP), phosphoenolpyruvate (PEP), ADP, ATP, NAD^+^, NADH, NADPH, KCN, pyruvate kinase, lactate dehydrogenase, cytochrome *c*, and decylubiquinone were from Sigma; KO was a generous gift of Aker BioMarine ASA (Oslo, Norway). Antibodies against AAC and UCP2 were from Santa Cruz Biotechnology (sc-11433 and sc-6526); antibodies against OXPHOS proteins were from Mitosciences (ab110413). Kits for the assay of triglycerides and total cholesterol were purchased from Futura System. Plasma insulin concentration was analyzed with a Mercodia Ultrasensitive Mouse Insulin kit. Luciferase ATP assay kit was from Sigma and Lipid Hydroperoxide (LPO) assay kit was from Merck. All other reagents were of analytical grade.

### 2.2. Ethics Statement

This study was carried out in strict accordance with the European Committee Council 106 Directive (86/609/EEC) and with the Italian animal welfare legislation (art 4 and 5 of D.L. 116/92). The Italian Ministry of Health specifically approved this study.

### 2.3. Animals and Diets

Male Sprague-Dawley rats (70–100 g) were obtained from Harlan (Carezzana, Italy) and housed individually in animal cages at a temperature of 22 ± 1°C with a 12 : 12 hour light-dark cycle and 30–40% humidity. After 1 week of acclimatization, 15 rats were divided into three groups of 5 animals each. The first group (control group) received a standard diet, also known as standard rodent chow, containing 6.2% fat and 44.2% carbohydrates (Global Diet 2018S from Harlan Teklad). The second group received a diet with a higher content (35%) of fat (and therefore referred to as HF group) and 36.1% of carbohydrates (Diet TD.03584 from Harlan Teklad). The third group of animals (HF+KO group) was fed with the above reported HF diet supplemented with 2.5% (w/w) KO. Diets composition is described in Table 1. The animals were treated for 4 weeks with* ad libitum* access to diets and water.

At the end of dietary treatment (after 4 weeks), body and liver weight were determined when rats were killed by decapitation, according to the guidelines for the care and use of laboratory animals. For the determination of plasma lipids, glucose, and insulin, control and treated rats were starved overnight before killing. Homeostasis model assessment (HOMA) index was calculated according to the following formula: HOMA = (fasting glucose (mM) × fasting insulin (*μ*U/mL))/22.5 [13].

Liver lipids were extracted and quantified as described in [14].

### 2.4. Assay of Enzymes Involved in Glucose and Fatty Acid Oxidation

Rat liver mitochondria were prepared by standard procedures and protein concentration was determined by the Bradford method [15].

Mitochondria (0.25 mg of mitochondrial protein/mL) were incubated in a buffer containing 25 mM MOPS and 0.05% Triton X-100 at pH 7.4. Solubilization of mitochondria with 0.05% Triton X-100 inhibited complex I of the respiratory chain preventing consumption of NADH. Pyruvate dehydrogenase (PDH) activity was measured spectrophotometrically as the rate of NAD^+^ reduction to NADH upon addition of 2.5 mM pyruvate, 0.1 mM CoA-SH, 0.2 mM TPP, 1 mM NAD^+^, and 5 mM MgCl_2_ (pH 7.4) [16].

Citrate synthase (CS) activity was determined in a medium containing 100 mM Tris-HCl (pH 8.1), 0.1 mM DTNB, and 0.25 mM acetyl-CoA. The reaction was started by adding 0.25 mM oxaloacetate, followed by monitoring changes at 412 nm for 3 min [17].

Total carnitine palmitoyl-CoA transferase (CPT) and CPT I activity was determined spectrophotometrically at 412 nm in freshly isolated rat liver mitochondria, essentially as described previously [18].

### 2.5. Mitochondrial Respiration Efficiency

Mitochondrial respiration (0.3 mg of mitochondrial protein/mL) was measured in a medium consisting of 220 mM sucrose, 20 mM KCl, 2.5 mM KH_2_PO_4_, 1 mM EDTA, 20 mM Hepes, 5 mM MgCl_2_, and 0.1% BSA, pH 7.4, by a Clark oxygen electrode at 25°C [2]. The addition of different substrates permitted to evaluate mitochondrial respiration when respiratory complexes I (5 mM pyruvate, 2.5 mM malate), II (5 mM K-succinate, 5 *μ*M rotenone), and IV (10 mM ascorbate, 0.2 mM TMPD, 5 *μ*M rotenone, 5 *μ*M antimycin A) were stimulated [19, 20]. Instead, the addition of 40 *μ*M palmitoyl-L-carnitine and 2.5 mM malate permitted revealing oxygen consumption from *β*-oxidation pathway.

For each substrate, after 2 min, state 3 respiration was induced by the addition of 0.3 mM ADP. Respiratory control ratio (RCR) was calculated as the ratio of the rate of oxygen uptake in the presence of added ADP (state 3) to the rate observed when added ADP had been completely phosphorylated to ATP (state 4).

### 2.6. Assay of Mitochondrial Respiratory Complex Activity

Complex I activity was determined using decylubiquinone as electron acceptor and NADH as donor. Rat liver mitochondria (40 *μ*g of protein) were pretreated first by freeze-thawing the samples two or three times in hypotonic medium consisting of 25 mM KH_2_PO_4_ (pH 7.2), 5 mM MgCl_2_ followed by a hypotonic shock in H_2_O to disrupt mitochondrial membranes. The reaction started after the addition of 50 mM Tris (pH 8.0) medium supplemented with 5 mg/mL BSA, 0.8 mM NADH as donor, 240 *μ*M KCN, 4 *μ*M antimycin A, and 50 *μ*M of the acceptor decylubiquinone. The oxidation of NADH was measured following the decrease in absorbance at 340 nm for 3 min [20].

Complex II activity assay was performed following the decrease in absorbance at 600 nm resulting from the reduction of DCPIP as electron acceptor and using succinate as donor. Rat liver mitochondria (40 *μ*g of protein) were pretreated first by freeze-thawing following by an incubation with succinate 10 min at 30°C in a medium containing 10 mM KH_2_PO_4_ (pH 7.8), 80 *μ*M DCPIP as acceptor, 4 *μ*M rotenone, and 0.2 mM ATP. The reaction started by addition of 80 *μ*M decylubiquinone and the activity was measured for 5 min [20].

Complex III activity was determined by measuring the reduction of oxidized cytochrome *c* at 550 nm. Rat liver mitochondria (40 *μ*g protein) were incubated for 1 min at 30°C in a reaction medium containing 50 mM KH_2_PO_4_ (pH 7.2), 0.01% Tween-20, 50 mM EDTA, 4 mM KCN, and 40 mM oxidized cytochrome *c*. The reaction was initiated by adding the ubiquinol analog, decylubiquinol (DBH_2_), to a final concentration of 50 *μ*M, and the rate of cytochrome *c* reduction was calculated from the absorbance increase at 550 nm [2].

Complex IV activity assay was performed following the decrease in absorbance at 550 nm resulting from the oxidation of reduced cytochrome *c*. Rat liver mitochondria (20 *μ*g of protein) were incubated in 1 mL of isosmotic medium containing 250 mM sucrose, 10 mM KH_2_PO_4_ (pH 6.5), and 10 *μ*M reduced cytochrome *c*. Cytochrome *c* solution was freshly reduced by adding some crystals of sodium dithionite. The addition of 2.5 mM lauryl maltoside permitted permeabilization of the outer mitochondrial membrane to cytochrome *c* and the decrease in absorbance at 550 nm was followed for 3 min [20].

ATP-synthase activity (ATP hydrolysis) was measured spectrophotometrically by a coupled assay using lactate dehydrogenase and pyruvate kinase as the coupling enzyme [20]. For each ATP molecule that is hydrolyzed, a molecule of NADH is oxidized. Rat liver mitochondria were incubated first in distilled water for 30 s at 37°C and then in a medium containing 200 *μ*L of 50 mM Tris (pH 8.0), 5 mg/mL BSA, 20 mM MgCl_2_, 50 mM KCl, 15 *μ*M CCCP, 5 *μ*M antimycin A, 10 mM PEP, 2.5 mM ATP, 4 units of lactate dehydrogenase and pyruvate kinase, and 1 mM NADH, previously incubated for 5 min at 37°C. Reaction was started by the addition of the medium to mitochondria and monitored for 3 min. Finally, the addition of 3 *μ*M oligomycin permitted distinguishing the ATPase activity coupled to the respiratory chain [20].

### 2.7. Western Blot Analysis

The expression of oxidative phosphorylation system (OXPHOS) proteins and mitochondrial carrier proteins AAC (ADP/ATP carrier) and UCP2 (uncoupling protein 2) was determined by western blotting analysis. Polyacrylamide gel electrophoresis was performed in the presence of 0.1% SDS (SDS-PAGE) according to standard procedures. The mitochondrial proteins that have been separated by SDS-PAGE were transferred to a nitrocellulose membrane. For the OXPHOS proteins, a cocktail directed against NDUFB8 (Complex I), SDHB (Complex II), COR2 (Complex III), COX1 (Complex IV), and ATP5A (ATP-synthase) was used at a dilution 1 : 1 × 10^1^ and the immunoreaction was carried out with monoclonal antibody by chemiluminescence. For AAC, UCP2, and porin (a protein used as a control) detection, antisera were used at a dilution of 1 : 1 × 10^3^ and the immunoreaction was carried out with polyclonal antibody by chemiluminescence.

### 2.8. ATP and Lipid Hydroperoxide (LPO) Levels

ATP levels in liver homogenates were determined by using the luciferase ATP assay kit. The concentration of ATP in the samples was calculated from interpolation of a standard curve prepared with known amounts of ATP. Results were expressed as nmoles of ATP per mg of protein.

Lipid peroxidation levels in liver samples were determined using a lipid hydroperoxide assay kit which measures the redox reactions with ferrous ions. LPO content was expressed as LPO per mg of protein.

### 2.9. Statistical Analysis

Experimental data represent the means ± SD. The data were analyzed by one-way ANOVA and a Tukey-Kramer* post hoc* analysis was used to detect significant differences between the means at a level of *P* > 0.05.

## 3. Results

### 3.1. Body and Liver Weight, Serum Parameters, and Liver Fat Content

As shown in Table 2, a significant increase in body weight was recorded in HF animals after 4 weeks of dietary treatment, in agreement with the high caloric content of the diet. Interestingly, the supplementation of the HF diet with 2.5% KO significantly prevented this effect. Instead, no statistically significant variations were found in the liver weight of animals fed with the different experimental diets.

The blood glucose concentration increased in both HF and HF+KO animals after 4 weeks of dietary administration, in comparison to control rats. Such an increase was less evident in the case of the HF+KO fed animals. A massive increase in the levels of insulin was revealed in the plasma of HF animals, in comparison to control rats. Interestingly, the KO supplementation significantly mitigated this increase. HF rats showed a marked increase in HOMA index, demonstrating typical insulin resistance induced by the high-fat diet. In the HF+KO animals, HOMA decreased, showing reduced insulin resistance induced by the addition of KO.

**Figure 1:**
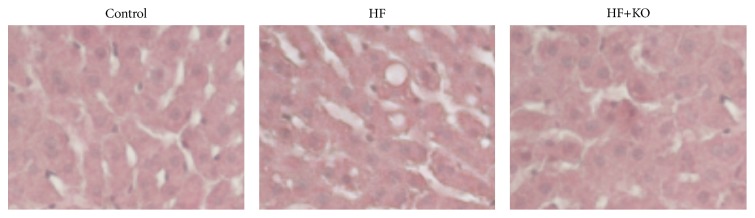
Liver samples were stored in neutral formaldehyde and embedded in paraffin wax. Sections (15 *μ*m) were stained with hematoxylin and eosin.

No significant difference was detected in the serum lipid (triglyceride and cholesterol) levels between the three groups of animals. On the contrary, liver lipid content was dramatically enhanced in HF animals, thus reflecting the presence of hepatic steatosis (Figure 1). The supplementation with KO of the HF diet reversed this effect, thereby keeping the lipid concentrations similar to those found in control rats. Accordingly, the liver histologic examination revealed a beginning of microvesicular fat depositions only in HF rats.

### 3.2. Pyruvate Dehydrogenase (PDH) Complex, Carnitine Palmitoyltransferase (CPT), and Citrate Synthase (CS) Activity

**Figure 2:**
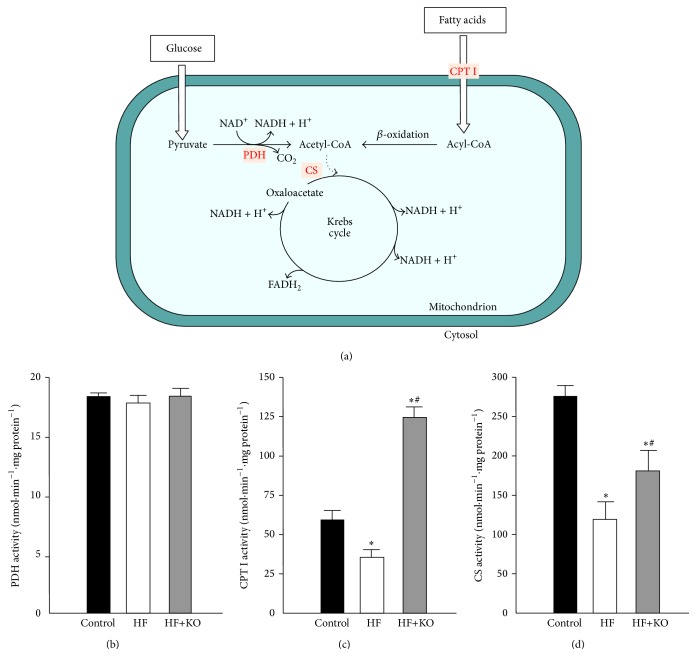
Mitochondrial metabolic pathways from glucose and fatty acid oxidation. Schematic picture of the metabolic pathways from glucose and fatty acid oxidation (a). Effect of KO on the activity of mitochondrial enzymes PDH (b), CPT I (c), and CS (d) in control, HF, or HF+KO-fed rats liver. The values reported in the figure represent the means ± SD (*n* = 5). ^*^ *P* < 0.05 versus rats fed a control diet; ^#^ *P* < 0.05 versus rats fed a HF diet.

Mitochondria play a central role in energy production. Sugars and fatty acids undergo glycolysis and mitochondrial *β*-oxidation, respectively, to produce acetyl-CoA (Figure 2(a)). PDH complex activity is the major determinant of glucose oxidation in animal cells. We found that PDH activity remained unchanged in the three groups of animals, suggesting that glucose catabolism was not affected by a HF diet or by KO supplementation (Figure 2(b)).

The limiting step for catabolic pathway of fatty acid oxidation is represented by the activity of CPT I, which is responsible for the entry of fatty acids into the mitochondrial matrix, where *β*-oxidation of fatty acids occurs. As shown in Figure 2(c), in the HF animals a clear decrease (about 40%) in the CPT I activity was detected. On the contrary, a strong increase in CPT I activity was found in the HF+KO rats. Thus, KO supplemented to the HF diet strongly stimulates fatty acid oxidation.

Acetyl-CoA, generated essentially from glucose or from fatty acids, is channeled into the Krebs cycle, where CS (the rate-limiting enzyme of the Krebs cycle) catalyzes its condensation with oxaloacetate to synthesize citrate (Figure 2(a)). A significant decrease (about 55%) in the activity of the CS was found in HF animals (Figure 2(d)), while only a moderate decrease (about 35%) was observed in HF+KO animals in comparison to the control groups. The partial reversal of CS inhibition observed in HF animals after KO supplementation could be explained by a compensatory response of this enzyme to sustain oxidation of acetyl-CoA generated from fatty acid oxidation.

### 3.3. Mitochondrial Oxygen Consumption, Respiratory Chain Complex Activities, and ATP Synthesis

The increased fatty acid oxidation observed in HF+KO animals produces higher levels of reducing equivalents, which are normally addressed towards the mitochondrial oxidative phosphorylation for ATP production. We therefore analyzed the respiratory efficiency of freshly isolated mitochondria by oxygraphic methods. Mitochondrial oxygen consumption was measured with various substrates in the presence (state 3) or the absence (state 4) of ADP (Table 3). According to the results shown in Figure 2(c), when we used palmitoyl-L-carnitine and malate as substrates, *V*_3_ (mitochondrial respiration state 3) decreased in HF animals thus indicating lower mitochondrial *β*-oxidation. On the other hand, the increase in the *V*_4_ (mitochondrial respiration state 4) values suggests that the excess of dietary fat most probably is the cause of a partial uncoupling between respiration and phosphorylation in mitochondria. Interestingly, the dietary KO supplementation is able to efficiently abolish this effect, keeping the mitochondrial respiratory efficiency unaltered.

When we added respiratory substrates for mitochondrial complexes I, II, and IV (Table 3), a little yet significant decrease in *V*_3_ was observed in the HF groups in comparison to control animals. In the HF+KO animals a significant increase in the *V*_3_ values was found in comparison to HF and control animals. *V*_4_, also known as the resting state of respiration, showed an increase in the HF and HF+KO group, even if the increase observed after KO supplementation was less evident than that found in HF animals. As a consequence, the RCR values were profoundly and significantly lower in HF animals with respect to those calculated for control animals. The KO supplementation of the HF diet reversed this effect, thereby keeping RCR values similar to those calculated for control group.

**Figure 3:**
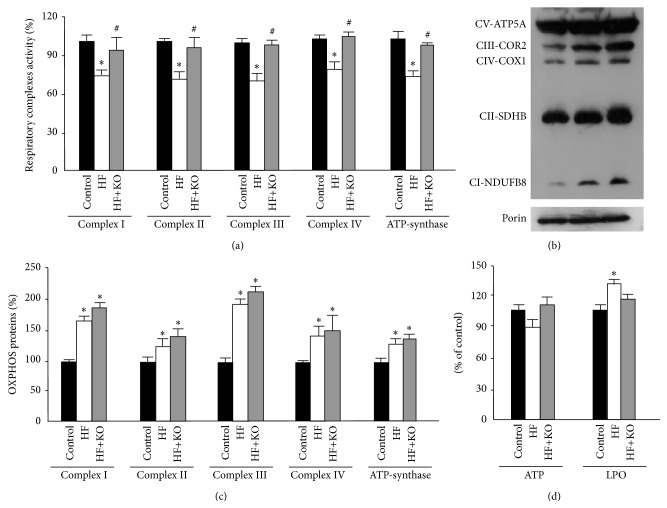
Effect of KO on respiratory complexes activity, expression, and function. Effect of KO on respiratory complexes activity (a). Enzymatic activities measured in the control group were set to 100%. Effect of KO on OXPHOS proteins levels (b). Liver mitochondrial proteins from control, HF, or HF+KO-fed rats were separated by SDS-PAGE, transferred to nitrocellulose, and then immunodecorated with a cocktail against NDUFB8, SDHB, COR2, COX1, ATP5A, or the mammalian porin as a control. The amount of OXPHOS proteins revealed by immunodecoration in the control group was set to 100% (c). ATP and LPO quantification of liver samples isolated from animals fed a control diet, a HF diet, or a HF+KO (d). The amount of ATP or LPO (expressed as nmoles per mg protein) in the control group was set to 100%. The values reported in the figure represent the means ± SD (*n* = 5). ^*^ *P* < 0.05 versus rats fed control diet; ^#^ *P* < 0.05 versus rats fed HF diet.

In a more selective approach for investigating the functionality of the mitochondrial oxidative phosphorylation we assayed the activity of single components of the respiratory chain: complexes I–IV and ATP-synthase (Figure 3(a)). A significant decrease (about 30%) in the activity of all respiratory chain complexes was found in HF animals. On the contrary, the values of complexes activity of the HF+KO rats were practically identical to those measured in control animals.

Surprisingly, western blotting analysis of the OXPHOS proteins showed significantly higher protein levels in both HF and HF+KO animals compared to the control group (Figures 3(b) and 3(c)). Such an increase was more evident for complexes I, III, and IV, which are associated as supercomplexes in the mitochondrial inner membrane [21]. This effect could be determined by a compensatory mechanism related to the reduction of the activities of the respiratory complexes.

In order to prove biological relevance of alteration in mitochondrial respiratory chain activity, we measured ATP concentration and oxidative damage in liver samples from the three groups of animals (Figure 3(d)). ATP quantification demonstrated that a HF diet caused a slight but not significant decrease in ATP content, an effect that was rescued by KO. Interestingly, in parallel experiments, we found a significant increase (about 30%) in the LPO levels in HF animals and this effect was efficiently reversed by KO supplementation of the HF diet.

### 3.4. Expression of Proteins Related to the Respiratory Chain Function

**Figure 4:**
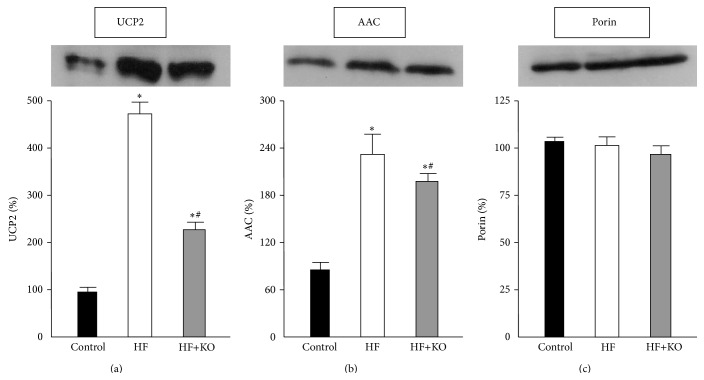
Effect of KO on proteins related to the respiratory chain function. Effect of KO on proteins levels of UCP2 (a) and AAC (b). The amount of porin, an outer membrane protein, was tested as a control (c). Liver mitochondrial proteins from control, HF, or HF+KO-fed rats were separated by SDS-PAGE, transferred to nitrocellulose, and then immunodecorated with antisera against UCP2, AAC or the mammalian porin. The amount of proteins revealed by immunodecoration in the control group was set to 100%. The values reported in the figure represent the means ± SD (*n* = 5). ^*^ *P* < 0.05 versus rats fed control diet; ^#^ *P* < 0.05 versus rats fed HF diet.

Uncoupling protein 2 (UCP2) uncouples respiration from oxidative phosphorylation. We therefore analyzed the expression profile of this carrier protein in the three groups of animals (Figure 4(a)). After 4 weeks of HF dietary treatment the amount of this protein increased about 5-fold, compared to the control group. Interestingly, a significant smaller increase in the expression of UCP2 was found after KO supplementation. In fact, in the presence of KO in the HF diet the level of this carrier protein was about 50% of that found in HF rats.

The AAC (ADP/ATP carrier) is another mitochondrial carrier which is able to regulate the basal proton conductance. This protein exchanges ADP for ATP across the mitochondrial inner membrane and may also play an important role in the mitochondrial permeability transition pore. When we analyzed the expression of this carrier protein (Figure 4(b)), we found an increase of about 2.2-fold in HF animals compared to the control group. AAC levels in the HF+KO animals, even if higher than those found in the control rats, were significantly lower than those found in HF group. The amount of porin, an outer membrane protein tested as a control, did not change in any group of animals (Figure 4(c)).

These results suggest that the KO supplementation is able to modulate the expression of mitochondrial carrier protein involved in the regulation of proton conductance, reducing the uncoupling effect caused by a HF diet.

## 4. Discussions

In animal experiments, a diet rich in fat or carbohydrates has been shown to induce hyperglycemia and hyperinsulinemia and to contribute to the development of obesity and NAFLD [2, 6, 7, 22]. This last condition is recognized as the most common chronic liver disease in the Western world. NASH is a more severe form of NAFLD that is broadly defined by the presence of steatosis with inflammation and progressive fibrosis, leading then to cirrhosis and hepatocellular carcinoma. Some patients with NAFLD develop NASH through poorly understood mechanisms. Since NAFLD and NASH are considered lifestyle-associated diseases, diet intervention is an important approach to their treatment.

In this context it is important to underline that the “obesogenic” Western diet has a ratio of fatty acids and carbohydrates of approximately 50/50. Moreover, it is characterized by an insufficient n-3 PUFAs consumption and a high n-6/n-3 ratio, which are associated with the progression from simple steatosis to NASH [8] along with modifications of gut microbiota [23].

Although the mechanisms responsible for increased lipid accumulation in the liver are not yet fully elucidated, decreased capacity to oxidize fatty acids, increased transport of fatty acids to liver, and increased hepatic fatty acid synthesis seem to be important factors involved in the pathogenesis of NAFLD [2, 6, 7, 24]. Moreover, abnormalities in mitochondrial morphology and functions seem to be associated with fatty liver [1–3, 25–27]. This finding suggests that regulation of mitochondria function and of molecular mechanisms involved in lipid accumulation in the liver could provide useful targets to prevent the development of NAFLD.

In recent years, the use of dietary supplements has rapidly increased [2, 6, 10, 24, 28] with the aim to prevent NAFLD. KO, a dietary supplement of n-3 PUFAs, has become increasingly popular because of its capability of reducing hepatic lipogenesis and of stimulating catabolization of excess fat introduced by a HF diet [2, 10]. The analysis of hepatic transcriptome in mice demonstrated that KO supplementation was able to regulate genes involved in hepatic energy metabolism, including glucose metabolism, lipid biosynthesis, fatty acid metabolism, cholesterol biosynthesis, and mitochondrial electron transport, more efficiently than fish oil [10, 12]. These differential results were tentatively ascribed to the influence of the structural form of n-3 PUFAs, esterified to either phospholipids or triglycerides, on a biological response. Moreover, PUFAs as phospholipids (KO) or triglycerides (fish oil) may differentially influence gut microbiota or synthesis of bacterial metabolites.

A previous study by our group [2] suggested that KO supplementation to a HF diet resulted in a retention of normal mitochondrial respiration efficiency, which was impaired in HF animals. The aim of the present study was therefore to analyze the molecular mechanisms by which dietary fats influence mitochondrial energetic metabolism. This new and intriguing aspect was evaluated in hepatocytes from rats fed a HF diet or a HF diet supplemented with 2.5% KO, with the aim to investigate the role of mitochondria in the modulation of nutrient metabolism exerted by KO.

No change in PDH activity, which essentially produces acetyl-CoA from carbohydrate, was seen after administration of HF or HF+KO diets. Nevertheless, fasting serum glucose and insulin were higher after feeding a HF diet, suggesting an altered insulin sensitivity as indicated by HOMA index changes. In fact, an excess of energy supplied by fat corresponds to a scarce tissutal utilization of glucose, thereby causing hyperglycemia and hyperinsulinemia. Insulin resistance was reduced after KO supplementation.

CPT I activity, which is involved in fatty acid oxidation, was inhibited by a HF diet and stimulated after KO supplementation. The increase in CPT I activity observed in HF+KO group could be explained by the lower levels of malonyl-CoA, a metabolic intermediate of hepatic lipogenesis, which, in this case, is instead strongly inhibited [2, 10]. Interestingly, the capability of KO to stimulate fatty acid oxidation seems to be associated to the presence of a high lipid content (35%) in the experimental diet used in this study. In fact, when KO was added to a standard diet, the expression of enzymes involved in mitochondrial fatty acid *β*-oxidation significantly decreased [12].

The modulation of the CPT I activity, observed in HF and HF+KO animals, seems to be accompanied by a parallel modulation of the activity of CS, a Krebs cycle enzyme that catalyzes the synthesis of citrate from oxaloacetate and acetyl-CoA. Recently, a strong and significant correlation of CS with obesity was noted, with a reduced enzyme activity in mitochondria of human omental adipose tissue in the state of obesity [28]. According to this observation, the increase in CS activity revealed after KO supplementation could be explained as a compensatory response to maintain the entry of acetyl-CoA produced by fatty acid oxidation into the Krebs cycle. It is interesting to underline that citrate generated by CS continues in the Krebs cycle. In fact, in animals fed a HF diet supplemented with KO a reduction in the activity and in the expression of citrate carrier protein (CIC), which transports citrate from mitochondria to the cytosol for fatty acid synthesis, was observed [2, 10, 24].

NADH and FADH_2_ generated by *β*-oxidation and Krebs cycle are used in the final common oxidative phosphorylation system (OXPHOS) to generate ATP. This process is coupled with the transfer of electrons along the respiratory chain. Our data indicate that a HF diet caused a strong decrease in the mitochondrial respiratory efficiency and this effect could be due to a possible uncoupling effect exerted by the excess of fatty acids present in this diet. In fact, we found a little decrease in *V*_3_ and a strong increase in *V*_4_ values in liver mitochondria isolated from HF animals. *V*_4_ is the resting state of mitochondrial respiration and an increase in this value suggests a stimulus of mitochondrial respiration independent of ADP phosphorylation. In other terms, during HF feeding, a lower coupling between mitochondrial respiration and ATP synthesis occurs. Interestingly, the addition of KO to the HF diet almost completely reversed this effect, thereby leading mitochondria respiration efficiency to be comparable to that of control rats.

When we assayed, in a more selective approach, the activity of single components of the respiratory chain, we observed a significant decrease in the activity of all respiratory chain complexes in HF animals, according to a recent study [29] in which an impairment of mitochondrial function and, in particular, of complex I activity was found. After KO supplementation, the values of complex activities were identical to those measured in control animals. Expression of OXPHOS proteins significantly increased in HF animals compared to the control group and this evidence was accompanied by the presence of ATP levels in HF animals that were only slightly decreased in comparison to those found in control animals. According to this result, previous studies [30, 31] demonstrated that a high fat content in the diet induced higher level of these proteins as a compensatory response to the reduced activity of the respiratory chain complexes. When we performed western blotting analysis in mitochondria isolated from HF+KO animals liver, we found a higher level of the OXPHOS proteins compared to the control and the HF groups, probably caused by the synergistic effect of the HF diet and the KO supplementation. Indeed, it is already known that genes involved in mitochondrial OXPHOS proteins expression were upregulated in mice consuming KO as revealed from the transcriptomic analysis [12]. Overall, this means that KO is able to induce the burning of excess of fat introduced by diet stimulating fatty acid oxidation, Krebs cycle, and respiratory chain complex activity and preventing a condition of oxidative damage.

Uncoupling between mitochondrial respiration and ATP synthesis, observed in HF animals, could be caused by a different expression of mitochondrial carrier proteins involved in the regulation of proton conductance. It is known that uncoupling proteins (UCPs) homologues uncouple mitochondrial respiration from oxidative phosphorylation, increasing thermogenesis while reducing the efficiency of ATP synthesis. In a previous study [32], an increase in the expression of the UCP2 isoform was found in rats with NAFLD. Interestingly, in this study we found a massive increase of this carrier protein in HF animals, but the addition of KO to the diet was able to strongly reduce the expression of hepatic UCP2. Therefore, it is reasonable to assume that the addition of KO to the HF diet can increase the coupling between mitochondrial respiration and ATP synthesis by a downregulation of UCP2 expression.

Not only are UCPs involved in mitochondrial uncoupling, but AAC is another carrier protein of particular interest in the understanding of the mitochondrial energy coupling process [33]. This carrier catalyzes the exchange of ADP^3−^ for ATP^4−^ and therefore utilizes, as its driving force, the electrochemical potential generated across the inner membrane by the respiratory chain. The exchange reaction is electrogenic and driven by the electrical component of the proton gradient, so that entry of ADP into mitochondria and exit of ATP are favored. We can therefore speculate that, if mitochondria are uncoupled and proton gradient was dissipated by the entry of H^+^ into the matrix by UCP2, an upregulation of the expression of AAC could compensate a reduced flux of ATP from mitochondria when membrane potential was reduced. This hypothesized compensatory mechanism was observed in animals fed a HF diet. The increase in the expression of AAC was less evident in HF+KO animals, in which liver mitochondria respiratory efficiency was unaltered in comparison to the control animals.

**Figure 5:**
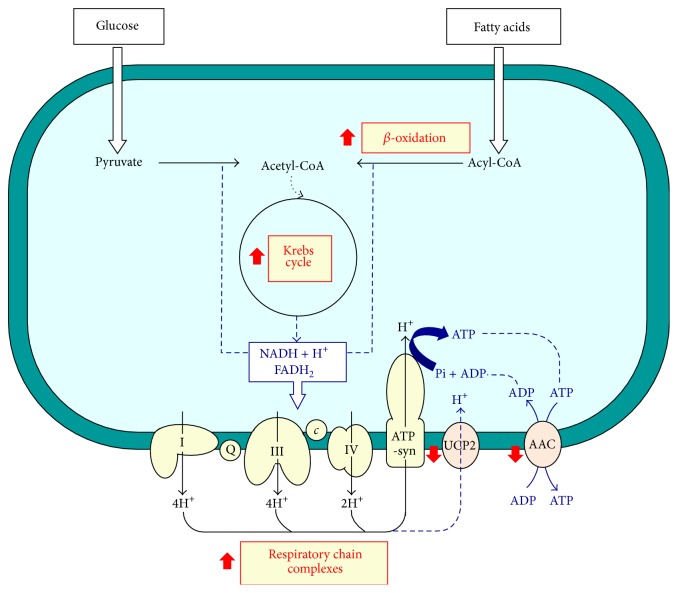
Mitochondrial pathways influenced by the addition of KO to a HF diet. *c*, cytochrome *c*; Q, ubiquinone.

In conclusion, the present study indicates that KO counteracts the negative effects of a HF diet. In particular, our results contribute to elucidate the molecular mechanism (Figure 5) by which KO positively influences many metabolic steps and ameliorates mitochondrial dysfunctions caused by the administration of a hyperlipidic diet, which often characterizes the nutritional habits of western populations. Nevertheless, additional studies in humans are required to confirm this view, since rodents may respond to the dietary macronutrients composition in a different manner than human subjects.

## Figures and Tables

**Table 1:** Composition of diets (%).

	Control	HF	HF+KO
Proteins	18.6	20.4	19.9
Lipids	6.2	35.2	36.8
Fatty acids			
14:0	—	0.5	0.6
16:0	0.7	8.7	9.0
18:0	0.2	4.3	4.3
16:1	—	—	0.1
18:1 (n9-7-5)	1.2	15.8	16.0
18:2 (n6)	3.1	3.5	3.5
18:3 (n3)	0.3	—	—
20:5 (n3) EPA	—	—	0.3
22:6 (n3) DHA	—	—	0.2
∑SFA	0.9	13.5	13.9
∑MUFA	1.3	15.8	16.1
∑PUFA	3.4	3.5	4.1
∑PUFA n-3	0.3	—	0.6
Carbohydrates	44.2	36.1	35.2
kcal/100 g	310	540	549
Calories from protein (%)	24.0	15.0	14.0
Calories from fat (%)	18.0	58.0	60.0
Calories from carbohydrates (%)	58.0	27.0	26.0

The control group of animals received a standard diet (Global Diet 2018S from Harlan Teklad). The HF group received a diet with 35% fat (Diet TD.03584 from Harlan Teklad) and the KO group was fed with the above reported HF diet supplemented with 2.5% (w/w) KO. Fatty acids were extracted from the three diets and analyzed by gas-liquid chromatography. Energy content was calculated using 4 kcal/g for protein and carbohydrate and 9 kcal/g for lipid.

**Table 2:** Body and liver weight, serum parameters, and liver fat content.

	Control	HF	HF+KO
Body weight (g)	245.8 ± 7.8	277.2 ± 15.8^*^	235.2 ± 25.2^#^
Liver weight (g/100 g body weight)	3.9 ± 0.6	3.5 ± 0.5	4.1 ± 0.3
Glycemia (mM)	2.9 ± 0.1	4.9 ± 0.7^*^	4.1 ± 0.4^∗#^
Insulin (*μ*U/mL)	50.1 ± 7.5	142.8 ± 10.0^*^	77.7 ± 5.0^∗#^
HOMA	6.4 ± 0.1	31.2 ± 0.3^*^	14.2 ± 0.1^∗#^
Serum triglycerides (mg/dL)	137.3 ± 12.9	147.1 ± 8.8	138.6 ± 9.2
Serum cholesterol (mg/dL)	92.6 ± 8.3	95.6 ± 4.2	94.1 ± 6.2
Liver triglycerides (mg/g liver)	8.6 ± 0.4	13.6 ± 1.0^*^	8.3 ± 0.5^#^
Liver cholesterol (mg/g liver)	2.7 ± 0.4	6.6 ± 0.4^*^	3.5 ± 0.5^#^

Each point represents the mean ± SD for 5 animals. ^*^ *P* < 0.05 versus rats fed a control diet; ^#^ *P* < 0.05 versus rats fed HF diet.

**Table 3:** Mitochondrial respiratory efficiency.

		Control	HF	HF+KO
Palmitoyl-L-carnitine + malate (*β* oxidation)	*V*_3_ (nmol O_2_·mL^−1^·min^−1^)	85.8 ± 7.8	59.7 ± 8.5^*^	114.0 ± 11.2^∗#^
	*V*_4_ (nmol O_2_·mL^−1^·min^−1^)	14.3 ± 2.4	22.9 ± 1.5^*^	18.5 ± 5.2^∗#^
	RCR	6.0 ± 0.4	2.6 ± 0.3^*^	6.1 ± 0.8^#^

Pyruvate + malate (Complex I)	*V*_3_ (nmol O_2_·mL^−1^·min^−1^)	17.0 ± 0.3	15.9 ± 0.4^*^	19.9 ± 0.9^∗#^
	*V*_4_ (nmol O_2_·mL^−1^·min^−1^)	5.4 ± 0.1	7.6 ± 0.2^*^	6.6 ± 0.6^∗#^
	RCR	3.2 ± 0.3	2.1 ± 0.3^*^	3.0 ± 0.4^#^

Succinate + rotenone (Complex II)	*V*_3_ (nmol O_2_·mL^−1^·min^−1^)	61.5 ± 3.3	53.1 ± 3.7^*^	78.3 ± 4.0^∗#^
	*V*_4_ (nmol O_2_·mL^−1^·min^−1^)	10.1 ± 0.3	25.0 ± 1.2^*^	15.0 ± 1.9^∗#^
	RCR	6.1 ± 0.9	2.1 ± 0.8^*^	5.2 ± 1.0^#^

Ascorbate + TMPD + rotenone + antimycin A (Complex IV)	*V*_3_ (nmol O_2_·mL^−1^·min^−1^)	55.1 ± 5.3	47.5 ± 6.5^*^	68.6 ± 7.8^∗#^
	*V*_4_ (nmol O_2_·mL^−1^·min^−1^)	9.3 ± 0.7	17.2 ± 0.9^*^	12.8 ± 0.8^∗#^
	RCR	5.9 ± 0.6	2.8 ± 0.3^*^	5.4 ± 0.9^#^

Respiratory control ratio (RCR) was calculated as the ratio of the rate of oxygen uptake in the presence of added ADP (*V*_3_) to the rate observed when added ADP had been completely phosphorylated to ATP (*V*_4_).

^*^ *P* < 0.05 versus rats fed control diet; ^#^ *P* < 0.05 versus rats fed HF diet.
